# Draft Genome Sequences of 13 Colombian *Helicobacter pylori* Strains Isolated from Pacific Coast and Andean Residents

**DOI:** 10.1128/genomeA.00113-17

**Published:** 2017-04-13

**Authors:** Alvaro Pazos, Nuri Kodaman, M. Blanca Piazuelo, Judith Romero-Gallo, Rafal S. Sobota, Dawn A. Israel, Luis E. Bravo, Douglas R. Morgan, Keith T. Wilson, Pelayo Correa, Richard M. Peek, Scott M. Williams, Barbara G. Schneider

**Affiliations:** a Division of Gastroenterology, Department of Medicine, Vanderbilt University Medical Center, Nashville, Tennessee, USA; b Department of Epidemiology and Biostatistics, Case Western Reserve University, Cleveland, Ohio, USA; c Vanderbilt Genetics Institute, Vanderbilt University Medical Center, Nashville, Tennessee, USA; d Department of Pathology, School of Medicine, Universidad del Valle, Cali, Colombia; e Department of Veterans Affairs, Veterans Affairs Tennessee Valley Healthcare System and Office of Medical Research, Nashville, Tennessee, USA

## Abstract

We present here the draft genomes of 13 *Helicobacter pylori* strains isolated from Colombian residents on the Pacific coast (*n* = 6) and in the Andes mountains (*n* = 7), locations that differ in gastric cancer risk. These 13 strains were obtained from individuals with diagnosed gastric lesions.

## GENOME ANNOUNCEMENT

Infection of human gastric mucosae with* Helicobacter pylori* is the major known risk factor for gastric cancer (1, 2), a disease that killed an estimated 723,000 people worldwide in 2012 and is the third most common cause of cancer deaths (3). Infection is typically acquired in childhood, and about half of the world’s population is infected. Although most infected persons have mild symptoms and no serious sequelae, a small proportion (1 to 3%) of those infected may develop gastric cancer. The series of lesions that may lead to the intestinal type of gastric cancer include nonatrophic gastritis, multifocal atrophic gastritis, intestinal metaplasia, and dysplasia (4, 5). Previously we reported that the disrupted coevolution of human hosts and *H. pylori* genomes is associated with more advanced gastric lesions in Colombian populations (6).

Here, we present draft genomes of 13 *H. pylori* strains isolated from residents of Tumaco (*n* = 6) on the Pacific coast, where incidence of gastric cancer is low, and from residents of Túquerres (*n* = 7) in the Andes mountains, where incidence is high. All participants provided informed consent; the study was approved by the institutional review boards of Vanderbilt University Medical Center and of the local hospitals. Participants were 40 years of age or older and were genotyped using the Immunochip (7), as previously described (6), to estimate ancestry (Table 1). Diagnosis of gastric biopsies and cultures of one antral gastric biopsy per subject were performed as previously described (6). DNA from the *H. pylori* pellet was isolated with DNAzol (Thermo Fisher Scientific) and then sheared and used to prepare a library for 250-bp paired-end sequencing with an Illumina MiSeq instrument. Sequencing reads were assembled *de novo* into contigs using CLC Genomics Workbench version 8.5 (CLC bio, Aarhus, Denmark). Calculated depth of coverage ranged from 36× to 142×. All genomes contained the *cag* pathogenicity island (8). Statistics for the assemblies are shown in Table 1. Draft sequences were annotated using the NCBI Prokaryotic Genome Annotation Pipeline.

**TABLE 1:** Statistics for Colombian *Helicobacter pylori* strains

Strain ID	Accession no.	Host residence	No. of contigs >200 bp	Coverage (×)	Genome size (bp)	*N*_50_ (bp)	Diagnosis	Age of host [yr(s)]	Estimated ancestry of host (%)		
									European	African	Amerind
PZ5005_3A3	MTWJ00000000	LR^a^	51	65	1,672,956	77,172	NAG^c^	53	16.4	76.5	17.1
PZ5006_3A3	MTWK00000000	LR	53	57	1,643,170	101,990	NAG	45	10	79.6	10.4
PZ5009_3A2	MSYO00000000	LR	53	101	1,677,035	96,381	NAG	53	22.7	60.8	16.5
PZ5016_3A3	MTWL00000000	LR	44	38	1,644,424	80,627	MAG^d^	40	19.6	49.1	31.3
PZ5019_3A3	MTWM00000000	LR	44	44	1,681,561	103,042	IM	47	23.4	29.7	46.9
PZ5033_3A2	MTWN00000000	LR	60	142	1,656,908	130,584	IM	57	11.8	72.6	15.5
SV328_2	MTWO00000000	HR^b^	56	37	1,645,479	95,218	Dys^e^	54	44.8	4.3	50.9
SV340_2	MTWP00000000	HR	53	41	1,633,298	128,372	NAG	45	16.6	0.8	82.6
SV355_2	MTWQ00000000	HR	39	41	1,635,304	125,154	IM^f^	45	13.1	1.8	85.1
SV376_1	MTWR00000000	HR	207	43	1,691,791	30,293	IM	55	45.6	7.6	46.8
SV380_1	MTWU00000000	HR	40	41	1,631,819	136,108	IM	43	36.9	0.7	62.5
SV397_2	MTWS00000000	HR	47	38	1,668,205	100,030	NAG^g^	45	32.8	1.4	65.8
SV449_1	MTWT00000000	HR	41	36	1,654,884	104,133	IM	42	28.3	9.2	62.5

a LR, host is resident of area where risk for gastric cancer is low.

b HR, host is resident of area where risk for gastric cancer is high.

c NAG, nonatrophic gastritis.

d MAG, multifocal atrophic gastritis.

e Dys, dysplasia.

f IM, intestinal metaplasia.

g Diagnosis from corpus biopsy only. Severity of lesions may be underestimated due to lack of incisura and antrum biopsies.

### Accession number(s).

The draft genome sequence projects presented here have been deposited at DDBJ/ENA/GenBank under the accession numbers shown in Table 1. The versions described in this paper are the first versions (e.g., MTWJ01000000 to MTWT01000000).
